# Targeting Amyloid Beta Aggregation and Neuroinflammation in Alzheimer’s Disease: Advances and Future Directions

**DOI:** 10.3390/cells15030295

**Published:** 2026-02-04

**Authors:** Ioanna Dagla, Faidon Gkikas, Evagelos Gikas, Anthony Tsarbopoulos

**Affiliations:** 1 Bioanalytical Laboratory, GAIA Research Center, The National Natural History Museum Goulandris, 145 62, Athens, Greece; ioanna.v.dagla@gmail.com (I.D.); bioan@gnhm.gr (F.G.); 2 Laboratory of Analytical Chemistry, Faculty of Chemistry, National and Kapodistrian University of Athens, 157 71 Athens, Greece; vgikas@chem.uoa.gr; 3 Department of Pharmacology, Medical School, National and Kapodistrian University of Athens, 115 27 Athens, Greece

**Keywords:** Alzheimer’s disease, natural products, curcumin, resveratrol, EGCG, cannabidiol, *Crocus sativus* L., neuroinflammation, inflammasome, amyloid-β aggregation, nanomedicine, nanoparticles

## Abstract

Alzheimer’s disease (AD) is a progressive neurodegenerative disorder and the most common cause of dementia in the elderly. Among the diverse pathological features of AD, amyloid beta (Aβ) aggregation and neuroinflammation are recognized as central and interlinked mechanisms driving disease progression. This review focuses specifically on these two processes and highlights current pharmacological limitations in modifying disease pathology. Natural products such as curcumin, resveratrol, *Ginkgo biloba*, epigallocatechin gallate (EGCG), crocin, ashwagandha, and cannabidiol (CBD) have shown promising activity in modulating Aβ aggregation and neuroinflammatory pathways, offering multi-target neuroprotective effects in preclinical studies. However, their therapeutic application remains hindered by poor solubility, instability, rapid metabolism, and limited blood–brain barrier (BBB) permeability. To overcome these barriers, nanotechnology-based drug delivery systems—including polymeric nanoparticles, niosomes, solid lipid nanoparticles, and chitosan-based carriers—have emerged as effective strategies to enhance brain targeting, bioavailability, and pharmacological efficacy. We summarize the mechanistic insights and nanomedicine approaches related to these bioactives and discuss their potential in developing future disease-modifying therapies. By focusing on Aβ aggregation and neuroinflammation, this review provides a targeted perspective on the evolving role of natural compounds and nanocarriers in AD treatment.

## 1. Introduction

Alzheimer’s disease (AD) is a neurodegenerative disorder representing the most common cause of senile dementia worldwide. AD primarily affects the brain’s structure and function, and it is characterized by a gradual decline in cognitive and non-cognitive function [1]. According to the Alzheimer’s Association, approximately 6.9 million Americans aged >65 years old are living with AD in 2024. The percentage of people with AD increases with age, and AD is the fourth main cause of death, affecting more than 36 million people worldwide, whereas it is projected to almost quadruple by 2050 [2]. Nevertheless, it is estimated that about 200,000 Americans aged 30–64 years have younger-onset dementia [3]. Approximately 35% to 60% of the early-onset AD cases (patients < 65 years old) are attributed to genetic factors, and the rest are classified as sporadic [4]. The risk of developing neurodegenerative diseases is steadily increasing dramatically due to increased lifespan, resulting in a significant burden for families, health-care systems, and society as a whole.

The precise pathophysiology of AD is not fully elucidated; however, several mechanisms that play a critical role in the disease progression have been proposed, such as protein aggregation, tau hyperphosphorylation, synaptic dysfunction, mitochondrial impairment, oxidative stress, and neuroinflammation [5]. The pathophysiology hallmarks of AD are deposits of extracellular amyloid beta (Aβ) peptide and accumulated hyperphosphorylated tau protein that form plaques and intraneuronal neurofibrillary tangles (NFTs), respectively [6]. Growing evidence indicates that the development of AD is not confined only to protein aggregation, but also involves significant interactions with the brain’s immune system [7]. Among the pathological mechanisms, Aβ aggregation and neuroinflammation stand out as two of the most important and interconnected processes driving disease initiation and progression [8]. These two mechanisms form a pathological feed-forward loop that exacerbates synaptic loss and neuronal death. Given the complexity of the disease, there is an increasing interest in the therapeutic strategies that simultaneously modulate both abnormal protein aggregation in the brain and neuroinflammation.

In general, AD represents one of the most remarkable scientific challenges for drug discovery, as the search for effective disease-modifying agents has been unsuccessful. In addition, immunotherapy treatment of AD by targeting the Aβ peptide has presented small benefits for people living with early Alzheimer’s, albeit with harmful risks (ARIA). Therefore, a full understanding of AD, including its pathological causes, prevention, management, and potential treatment, is needed. This review focuses exclusively on Aβ aggregation and neuroinflammation and presents an up-to-date overview of the modulators of amyloid beta aggregation and neuroinflammation, highlights the current trends, and discusses the future directions for the prevention and potential treatment of AD.

## 2. Methodology

*Study Design and Search Strategy*: This review was performed by a comprehensive literature search using PubMed, Web of Science, and Scopus as electronic databases. The search strategy targeted studies published up to July 2025, with a specific focus on recent advancements in nanomedicine between 2020 and 2025 to ensure the inclusion of the most current therapeutic trends. As search keywords were used combinations of: “Alzheimer’s disease,” “Amyloid beta aggregation,” “Neuroinflammation,” “NLRP3 inflammasome,” “Natural products,” “Curcumin,” “Resveratrol,” “EGCG,” “Saffron,” “Ashwagandha,” “Cannabidiol,” and “Nanomedicine/Nanoparticles”.

*Inclusion and Exclusion Criteria*: The inclusion criteria were either peer-reviewed original research articles and reviews or studies published in the English language. During the search process, those papers were judged to be most relevant to the focus of this Review. Abstracts, conference proceedings, or editorials without full-text availability were excluded.

*Quality Assessment and Data Synthesis*: While a formal statistical meta-analysis was not performed, the authors prioritized studies published in high-impact, peer-reviewed journals in order to estimate potential bias and ensure the quality of outcome measures.

*Limitations*: First, the quality of the reviewed studies is variable. A substantial proportion of the available evidence is derived from in vitro and animal models, which may not fully summarize the complexity of human AD. Most of the efficacy data presented are derived from in vitro assays and preclinical animal models (e.g., APP/PS1 mice). Despite their utility, these models do not fully replicate the complex, multifactorial pathophysiology of human AD. In addition, differences in experimental design, disease models, dosing regimens, and outcome measures limit direct comparison across studies and reduce applicability to clinical settings.

Second, the literature on natural products and nanoparticle-based delivery systems in AD remains heterogeneous and, in many cases, preliminary. On the other hand, as a review, it focuses on selected high-potential bioactive compounds and may be subject to selection bias, not incorporating the entire pharmacopeia of neuroprotective agents. Furthermore, a significant heterogeneity exists across the included studies with regard to the extraction methods, purity, and dosage of the natural products. Finally, while nanotechnology offers a solution to bioavailability issues, data concerning the long-term safety, biodegradability, and potential toxicity of these nanocarriers in the human brain remain insufficient, highlighting a critical gap that requires toxicological evaluation in future research. Consequently, conclusions regarding the potential therapeutic potential should be interpreted with caution.

## 3. Amyloid Beta Aggregation and Neuroinflammation in AD

**Figure 1:**
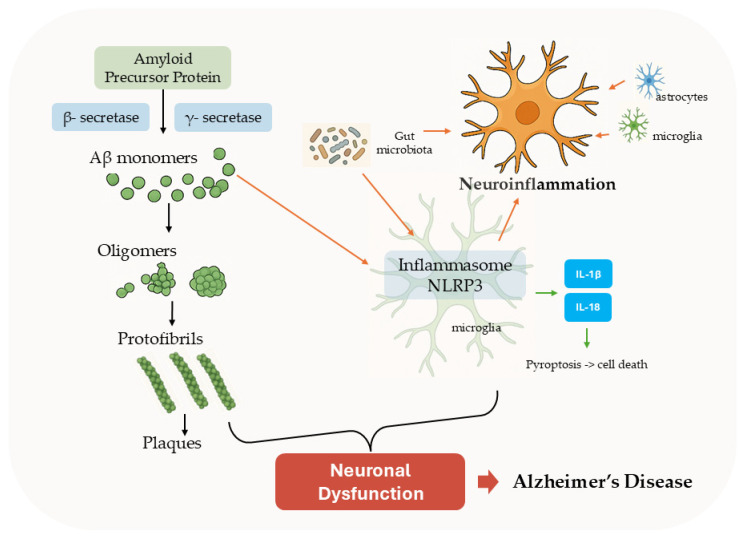
Illustration of Aβ aggregation and neuroinflammation mechanisms of AD pathogenesis.

AD develops through two main processes: the abnormal accumulation of misfolded Aβ peptide leading to the formation of senile plaques in the brain and ongoing inflammation of nerve system. Together, they damage neurons and drive the progression of the disease. The mechanisms of AD pathogenesis are presented in Figure 1.

### 3.1. Amyloid Beta Aggregation in AD

A hallmark feature in AD pathogenesis is the formation of amyloid plaques in the brain of patients. The amyloid plaques are formed by the abnormal extracellular accumulation of Aβ peptide that is generated from the cleavage of a larger protein, called amyloid precursor protein (APP). In the non-amyloidogenic pathway, APP is cleaved by membrane-bound enzyme α-secretase within its Aβ domain, resulting in the release of extracellular secretion of soluble α-secretase-cleaved sAPPα fragments. In the amyloidogenic pathway, APP is cleaved sequentially by two enzymes, β-secretase (also known as BACE1) and γ-secretase, resulting in the release of the amyloid intracellular domain (AICD) and the extracellular secretion of soluble Aβ. Therefore, any modifiers that can enhance the activity of α-secretase can reduce the production of neurotoxic Aβ and may prove beneficial towards the prevention and/or treatment of AD. The most extensively investigated Aβ forms are the Aβ_1–40_ and Aβ_1–42_ peptides. The Aβ_1–42_ isoform is much more prone to aggregation and more toxic to neurons than the Aβ_1–40_ isoform [9]. In healthy individuals, Aβ peptides are cleared efficiently from the brain, but in AD, the balance between production and clearance is disrupted, resulting in the accumulation of this peptide. The aggregation of Aβ monomers into soluble oligomers, then into protofibrils, and eventually into insoluble fibrils and plaques represents a critical pathogenic event in the amyloid cascade hypothesis of AD [10].

Soluble Aβ oligomers are considered the most neurotoxic species, as they induce neurotoxicity through several pathogenic mechanisms, including mitochondrial dysfunction, oxidative stress, calcium dysregulation, and disruption of synaptic signaling. These oligomers can also form ion channel pores in neuronal membranes, disrupting the intracellular calcium homeostasis and thus resulting in mitochondrial dysfunction and oxidative stress. Furthermore, Aβ oligomers interfere with hippocampal long-term potentiation and bind to cellular prion protein, leading to the disruption of synapses [11,12,13].

### 3.2. Neuroinflammation

Inflammation is defined as an innate response of the immune system, providing protection and defense in the body against harmful stimuli [14]. It results from either an aseptic insult, such as mechanical tissue injury, or a non-aseptic response to an infection of pathological origin. This innate response is described as a multifactorial process, characterized by the coordination and synergy of several cell types. These cells utilize signaling molecules to produce both local and systemic responses [15].

The protective mechanism resulting from inflammatory responses in brain cells is called neuroinflammation, a process related to the onset of various neurodegenerative diseases [16]. Neuroinflammation plays a prominent role in both the onset and progression of AD, involving a plethora of factors that contribute to the occurrence of neuronal dysfunction and death in AD, either alone or in combination [17,18].

AD is considered the most common neurodegenerative disease provoked by the extracellular deposit of Aβ plaques, intraneuronal NFTs of hyperphosphorylated tau protein, and neuroinflammation [19,20]. There is growing evidence that Aβ accumulation stimulates tau phosphorylation and fibrillary tangle formation, leading to the process of neurodegeneration [21]. Neuroinflammatory processes, primarily driven by immune cells, including glial cells and astrocytes, strongly affect neuronal function and survival [16]. Astrocytes and microglial cells are the primary cell types involved in the CNS inflammatory response [22].

It is a complex response involving not only the host’s brain cells and peripheral immune cells, but also molecular changes or induction of some intracellular signaling pathways and release of inflammatory mediators. This inflammatory response disrupts the neuronal microenvironment, leading to neuronal damage. Over time, these processes contribute to oxidative stress and promote cellular apoptosis, ultimately driving the onset of symptoms characteristic of AD [17,23].

On the other hand, it has recently been found that gut microbiota also affect the induction of neuroinflammation [16]. They interfere with metabolic homeostasis by producing and excreting amyloid, lipopolysaccharides, and other toxins which travel through the bloodstream and enter the brain, disrupting the blood–brain barrier, or via various pro-inflammatory mediators. Thus, the activation of microglia enhances the inflammatory response in the CNS [24]. Furthermore, gut microbiota activate the NLR family pyrin domain-containing 3 (NLRP3) inflammasome, and it induces the production of pro-inflammatory factors, including interleukins, which further increase inflammatory response, leading to AD [25].

AD is a progressive neurodegenerative disorder defined by characteristic histopathological and widespread structural brain deterioration. The two principal pathological features include: (i) focal extracellular deposits of fibrillar Aβ peptide —commonly referred to as neuritic or senile plaques—accumulating in the brain parenchyma and along cerebral blood vessel walls, and (ii) intraneuronal aggregation of neurofibrillary tangles (NFTs) composed of abnormally hyperphosphorylated tau protein filaments [26]. These pathological processes of disease initiate in the transentorhinal cortex and progressively extend to the entorhinal cortex and hippocampus, regions essential for memory and learning. As the disease advances, neurodegeneration spreads to other cortical areas, including the temporal, frontal, and parietal lobes, leading to widespread cortical involvement, resulting in extensive neuronal and synaptic loss, accompanied by global atrophy of the brain [27].

Several studies have shown that β-amyloid deposits present in the brains of AD patients drive the inflammation process due to the reduced concentration of neuroinflammation suppression mediators. β-Amyloid fibrils trigger neuroinflammation by accumulating in the brain and binding to the C1 component of the complement system [23]. This interaction activates the complement cascade, stimulating resident CNS immune cells (e.g., microglia and astrocytes) to mount an inflammatory response against β-amyloid deposits [22]. Notably, complement activation requires β-amyloid to be in a fibrillated state, highlighting the role of protein aggregation in driving neuroinflammatory pathways in AD, driving the inflammation process and causing its permanent maintenance [23].

In the central nervous system (CNS), defense is coordinated by the recruitment of microglia, astrocytes, and macrophages, but also oligodendrocytes, neurons, and endothelial cells participate. The detection of pathogenic agents is mediated by pattern recognition receptors (PRRs) that recognize pathogen-associated molecular patterns (PAMPs) and host- or environment-derived danger-associated molecular patterns (DAMPs) [7]. PRRs can be either membrane-bound, as with Toll-like receptors (TLRs), which perceive signals in the extracellular environment or endosomes, or intracellular, as with nucleotide-binding domain and leucine-rich repeat-containing receptors (NLRs) [28].

#### 3.2.1. Microglia

Microglia are mesenchymal, myeloid mononuclear resident phagocytes of CNS, and they are considered to have a principal role in the immune system response. They represent about the 10% of the neural cell population, and they play a crucial role in neurogenesis, neuronal plasticity, regeneration, and immune defense during brain injury [7,29,30]. The presence of extracellular Aβ deposits, NFTs of hyperphosphorylated tau protein (pTau), and inflammation is considered the hallmark of AD [22]. Aβ deposits and tau proteins are located in different brain regions, resulting in synaptic impairment, mitochondrial damage, activation of microglia, and neuronal death. The inflammatory response in AD is marked by reactive microglia surrounding Aβ plaques, which sustain an inflammatory state by releasing proinflammatory mediators, thereby contributing to neuronal damage [31].

Microglia have been found closely associated with Aβ plaques in the brains of individuals with Alzheimer’s disease (AD). One of their recognized beneficial roles in AD pathology is their ability to limit plaque formation through the clearance of toxic Aβ aggregates. As part of an early protective response, Aβ triggers chemoattraction, activation, and proliferation of microglia, helping to prevent further accumulation of Aβ [32]. In AD tau pathology, microglia try to clear toxic tau seeds through phagocytosis. Nevertheless, this phagocytic action is reduced at later stages of AD due to an uncoupling of microglia activation and phagocytosis functions, which can accelerate the spread of tau and the progression of neurodegeneration in AD [33].

Microglia are involved in homeostasis and host defense mechanisms against pathological infections or CNS disorders. In the context of their macrophagic action, they can phagocytose toxic substances, release cytotoxic factors, and present antigens. At the same time, when there isn’t any risk factor, microglia are dormant; they have a ramified morphology and can surveil their environment without disrupting neuronal activity, monitoring the brain parenchyma regularly [30,32]. Upon activation, they undergo morphological changes to an amoeboid, mobile form that can migrate to injury sites. Activated microglia may persist for extended periods, releasing cytokines and neurotoxic agents that contribute to long-term neurodegeneration and can exacerbate CNS damage [15].

There are three essential functions that microglia participate in. First, they scan their surrounding environment and detect potential changes mediated by sensomes, a group of genes that code for proteins expressed by microglia, allowing them to sense and respond to their environment [29,34]. On the second function, microglia migrate to injured sites where they are involved in synaptic remodeling. In the third function, they play a critical role in maintaining myelin homeostasis by recognizing danger-associated molecular patterns (DAMPs) and pathogen-associated molecular patterns (PAMPs), thus preventing CNS injury stimuli. The association of Aβ with the microglial receptors, including TREM2, TLRs, CD36, class A1 scavenger receptors (SR-A1), and receptor for advanced glycation end products (RAGE), activates microglia and mediates Aβ phagocytosis/endocytosis [35,36]. Upon activation, microglia produce pro-inflammatory cytokines such as tumor necrosis factor-alpha (TNF-α), interleukin-1β (IL-1β), IL-16, and chemokines, stimulating the recruitment of additional cells to confront pathological agents [29,37]. Although neuroinflammation serves as a neuroprotective mechanism, prolonged or chronic neuroinflammation can lead to deterioration of neuronal function, neurotoxicity, and ultimately neuronal death [37].

#### 3.2.2. Astrocytes

Astrocytes (also known as astroglia) are star-shaped glial cells, part of a class of neural cells (also known as astrocytes) of ectodermal, neuroepithelial origin [38]. Astrocytes, the most abundant glial cell type in the central nervous system, regulate neurotransmitter and calcium homeostasis, modulate synapse formation, maturation, and elimination, and play a pivotal role in maintaining neuronal function and homeostasis [39]. Generally regarded as supportive elements, but according to the latest evidence, astrocytes are increasingly recognized as active modulators of neurodegenerative processes, particularly in AD.

Astrocytes represent significant heterogeneity in both form and function, displaying remarkable adaptive synaptic plasticity that is essential for maintaining central nervous system (CNS) homeostasis throughout aging [38]. They modulate extracellular ion and fluid balance, contribute to the removal of free radicals, and play a critical role in supporting blood–brain barrier integrity and provide nutritional and trophic support to the brain [40,41].

Astrocytes have a dynamic and pathology-specific response to AD, known as reactive astrogliosis. This is a multistage process that participates in neuroprotection and tissue repair [41]. Alongside microglia, reactive astrocytes accumulate around Aβ plaques, form a physical barrier called glial scar, thus reducing collateral damage due to neurotoxicity [40]. Notably, glial activation may even precede Aβ deposition, suggesting astrocytic involvement in early disease mechanisms. Simultaneously, the accumulated reactive astrocytes have an increased expression of glial fibrillary acidic protein (GFAP) and vimentin, transforming them into hypertrophic cells [7]. Also, there are indications that reactive astrocytes clear or reduce Aβ deposits by phagocytosis, while astrocytes also participate in neuroinflammatory signaling, releasing cytokines, interleukins, nitric oxide, and other cytotoxic factors in response to Aβ, which amplify local inflammation [7].

On the other hand, many scientists support that reactive astrocytes are contributing to the presence of neurotoxicity by secreting significant quantities of Aβ and contributing to the overall amyloid burden in the brain [42]. Astrocytes, like microglia, detect Aβ aggregates using a dependent mechanism on TLR/RAGE, which triggers the expression of downstream target genes and the subsequent release of increased production of neurotoxic factors, leading to increased Aβ load and toxicity [40]. It should be noted that in preclinical AD, microglia and astrocytes become reactive early, even before symptoms arise, playing crucial roles in the disease’s onset and progression. Microglial and astrocyte biomarkers measured through fluid biomarkers increase early during asymptomatic stages of AD and have a significant influence on key pathogenic events at the preclinical stage. In particular, GFAP influences amyloid accumulation, whereas sTREM2 promotes tau pathology, thus contributing to the progression of neurodegenerative changes in the early stages of AD [43].

#### 3.2.3. Inflammasome

Inflammasomes constitute a valuable arm of the innate immunity, since they are inducible high molecular weight cytosolic multi-protein complexes that are involved in many inflammatory pathological processes [44]. These multimeric protein complexes are expressed in microglia, the principal cells in the innate immune system in the central nervous system [20].

Inflammasomes are separated into canonical and non-canonical inflammasomes. In the canonical category, they can control the activation of the proteolytic enzyme caspase-1. In contrast, non-canonical inflammasomes are characterized by the action of caspase-4 (corresponding to the murine caspase-11) and caspase-5 [45,46].

The inflammasome has a crucial role in initiating inflammation. It detects harmful stimuli such as pathogens, cellular stress, or damage signals and is mediated by pattern recognition receptors (PRRs) in microglia [47]. Pattern Recognition Receptors (PRRs) can be found either on the cell membrane or inside the cell. Membrane-bound PRRs, such as Toll-like receptors (TLRs), detect signals in the extracellular space or within endosomes, while intracellular PRRs, including members of the NLR (nucleotide-binding domain and leucine-rich repeat-containing receptors—LRRs) and ALR (AIM2-like receptors) families, are located within the cytoplasm. A key subgroup of these cytosolic PRRs, which encompasses NLRs (NOD-like receptors), ALRs (AIM2-like receptors), and the pyrin protein from the tripartite motif (TRIM) family [28,48].

Aβ protofibrils but also small Aβ oligomers were found to activate the NLRP3 inflammasome in microglial cells through multiple signaling pathways, including NF-κB, thereby defining a novel pathway that could lead to progression of AD [44]. Upon activation, the inflammasome regulates the proteolytic process, promoting the production and release of pro-inflammatory cytokines like interleukin-1β (IL-1β) and IL-18, and induces pyroptosis, which is a rapid, inflammatory form of lytic programmed cell death [49]. There are different types of inflammasomes, with the NLRP3 isoform being one of the most studied. The NLRP3 isoform appears to be largely responsible for promoting chronic inflammatory diseases, including atherosclerosis, diabetes, and neurodegenerative diseases such as AD [47,49]. NLRP3 inflammasome activation is a key driver in AD pathogenesis, linking Aβ and tau pathology to chronic inflammation by triggering the extracellular release of downstream cytokines, such as IL-1β, IL-10, and IL-18 from microglia and astrocytes. Microglial dysfunction by NLRP3 inflammasome signaling deteriorates the clearance capability of NFTs by microglia, and eventually sets up a proinflammatory cycle, accelerating neuronal cell death [50].

In AD, the NLRP3 inflammasome is like a control switch, i.e., its activation triggers an inflammatory response that harms neurons. Therefore, there is active research to explore ways to inactivate the NLRP3 inflammasome using drugs and natural products to suppress neuroinflammation and the ensuing responses [51]; thus, aiding microglia to clear the harmful Aβ deposits (i.e., phagocytosis). For example, the potential of nonsteroidal anti-inflammatory drugs (NSAIDs) in delaying the onset of AD is likely through their modulation of the NLRP3 inflammasome pathway. Similarly, natural products including flavonoids (e.g., quercetin) and polyphenols (curcumin, resveratrol) have demonstrated the ability to inhibit Aβ aggregation as well as the pro-inflammatory NLRP3 inflammasome.

## 4. FDA—Approved Therapeutics for Alzheimer’s Disease: Current Status and Limitations

According to Alzheimer’s Association (https://www.alz.org/alzheimers-dementia/treatments, accessed on 14 July 2025), two main classes of drugs for the AD treatment have been approved by FDA: (1) Disease-modifying therapies and (2) symptomatic treatments that are presented in Table 1. Anti-amyloid monoclonal antibodies, including donanemab (Kisunla™, Eli Lilly, Indianapolis, IN, USA) and lecanemab (Leqembi^®^, Eisai Co., Tokyo, Japan), are currently approved for clinical use as disease-modifying treatments for AD. These anti-amyloid monoclonal antibodies are administered intravenously and target the toxic form of Aβ aggregates (e.g., oligomers, protofibrils, fibrils, and plaques). They demonstrate efficacy in early stages of AD, reducing Aβ plaques and mitigating cognitive and functional decline, without stopping or reversing the progression of AD. Nonetheless, they are associated with health risks such as amyloid-related imaging abnormalities (ARIA) that cause headache, dizziness, nausea, confusion, and vision changes requiring patient monitoring. The future direction of next-generation anti-amyloid monoclonal antibodies is to proceed towards structure modifications of antibodies to minimize adverse effects, i.e., risk of ARIA, as well as enhance BBB permeability and therapeutic efficacy for the treatment of AD.

Symptomatic treatments include cholinesterase inhibitors, namely benzgalantamine, donepezil, galantamine, and rivastigmine, and the glutamate regulator memantine, targeting cognitive symptoms like memory and thinking. Combination therapy (donepezil plus memantine) is approved for moderate-to-severe AD. Non-cognitive symptoms, such as insomnia and agitation, are addressed by suvorexant and brexpiprazole, respectively. Moreover, there are therapeutic approaches addressing tau pathology, including anti-phosphorylation strategies and aggregation inhibitors. The former comprises the use of tau kinase inhibitors such as glycogen synthase kinase-3β (GSK-3β), whereas the latter involves the use of derivatives of methylene blue (e.g., Rember™, TauRx Therapeutics Ltd., Singapore). In addition, a second-generation tau aggregation inhibitor called TRx0237 (LMTX™) for the treatment of AD is currently in clinical trials. It is obvious that the existing pharmacological treatments mostly offer modest symptomatic relief or delayed progression, underscoring the need for further development of more effective therapies that target the underlying disease mechanisms and improve the life quality of AD patients.

## 5. Natural Products as Modulators of Amyloid Beta Aggregation and Neuroinflammation

Although FDA-approved medications for AD are available, these fail to modify the underlying disease processes, such as Aβ aggregation and neuroinflammation, offering only symptomatic relief. This therapeutic limitation has driven increasing interest in natural products, which exert multi-targeted actions and may modulate both pathological hallmarks [52,53].

In this review, we have focused on a selected group of natural products that perform dual modulatory effects on Aβ aggregation and neuroinflammatory pathways that are critical to AD pathogenesis. The natural products that are discussed in this review are summarized in Table 2. First, some natural products, such as Epigallocatechin-3-gallate (EGCG) and the omega-3 fatty acids DHA and EPA, have been reported to enhance the α-secretase non-amyloidogenic pathway, thus reducing the production of neurotoxic Aβ. On the other hand, several other natural products have demonstrated neuroprotection mainly due to their antioxidant and anti-inflammatory properties. These natural substances have been extensively studied in both in vitro and in vivo models for their ability to inhibit Aβ aggregation, disaggregate plaques and fibrils, or reduce oligomer toxicity. Concurrently, they exhibit anti-inflammatory activity through suppression of microglial activation and reduction of pro-inflammatory cytokine release. Obviously, these natural products are also involved in more AD pathogenic pathways, acting pleiotropically. Their natural origin, relative safety, and multi-targeted actions make them compelling candidates for AD disease treatment, especially within the context of Aβ aggregation and neuroinflammation.

### 5.1. Curcumin

Several experimental studies have demonstrated the neuroprotective effect of curcumin and its novel formulations in AD. Curcumin, the main polyphenol of *Curcuma longa* L., prevents or mitigates the neurodegenerative processes through a wide range of beneficial properties such as antioxidant, anti-inflammatory, and neurotrophic activities [54]. Mechanistically, curcumin inhibits the formation of Aβ protein, promotes the disaggregation of amyloid plaques, decreases the tau hyperphosphorylation, and accelerates the tau clearance [55,56]. Furthermore, it modulates microglial activity, inhibits acetylcholinesterase, regulates insulin signaling pathways, and binds metal ions such as copper [56]. Structural features such as phenyl methoxy groups and flexible moieties have been associated with enhancing curcumin’s efficacy by suppressing Aβ aggregation and APP expression [57]. Moreover, curcumin has been shown to advance the adult neurogenesis of AD mice by targeting the PI3K/Akt that controls GSK3β/Wnt/β-catenin and CREB/BDNF pathways [58]. Although these promising findings exist, curcumin’s clinical application is limited by poor bioavailability and suboptimal pharmacokinetics. Still, structural modifications and novel delivery strategies have shown likely to overcome these challenges, suggesting that curcumin-based therapeutics could offer a more effective alternative to current AD treatments, if bioavailability issues are overcome [56,59].

### 5.2. Resveratrol

Many studies have indicated resveratrol, a polyphenol that naturally occurs in red wine and other plants, as a promising neuroprotective product in AD [60] due to its anti-amyloidogenic, antioxidant, anti-inflammatory, and anti-aging properties [61]. Resveratrol is involved in Aβ pathogenesis as it inhibits Aβ aggregation, promotes non-amyloidogenic processing of the APP, and enhances Aβ clearance [61,62,63,64]. Furthermore, it decreases tau hyperphosphorylation and glial activation and promotes hippocampal neurogenesis [62]. Resveratrol can penetrate the blood–brain barrier, and thus, it enhances endogenous antioxidant enzymes and activates silent information regulator-1 (SIRT1) that plays a crucial role in growth, differentiation, and apoptotic death of neurons by suppressing p53 activity [61,62]. Recent meta-analyses have revealed that resveratrol’s neuroprotective properties are associated with the PI3K-Akt signaling pathway [65]. Still, the therapeutic ability of resveratrol in humans remains limited because of poor bioavailability and rapid metabolism; thus, novel delivery strategies and structural optimization are needed [64,66].

### 5.3. Ginkgo biloba

*Ginkgo biloba* extract (GBE), and mainly its standardized extract EGb 761, demonstrates promising neuroprotective effects in AD. Clinical studies show that GBE alleviates AD symptoms in early-stage patients after high doses and long-term administration [67]. GBE enhances memory and cognitive functions by mitigating Aβ-induced neurotoxicity via several ways, including metal dyshomeostasis, redox imbalance, and mitochondrial dysfunction. In addition, EGb 761 enhances cellular resistance to oxidative stress, thereby helping to protect neurons from oxidative damage commonly linked to AD [68]. Its antioxidant actions are associated with its impact on the cerebral blood flow, the nitric oxide level, the cellular redox stage, and the neurotransmitter system [69]. Furthermore, EGb 761 supports mitochondrial integrity, reducing apoptosis and promoting hippocampal neurogenesis. GBE also influences microglia phenotypes, as it reduces pro-inflammatory cytokines and upregulates anti-inflammatory markers such as IL-4, IL-13, and TGFβ, thus exerting an anti-inflammatory effect [69]. Meta-analysis results have shown that *Ginkgo biloba* preparations combined with donepezil can improve clinical effectiveness and cognitive performance without a change in adverse reactions [70]. Due to inconsistent results across several clinical trials, it has been emphasized that there is a need for further research to clarify critical aspects such as the optimal dosing, the delivery methods, the formulation, and potential adverse effects of GBE [71,72].

### 5.4. Epigallocatechin Gallate—EGCG

Epigallocatechin-3-gallate (EGCG), a natural catechin and the main bioactive compound of green tea with potent antioxidant properties, has shown significant therapeutic promise in AD. Several epidemiological studies have indicated the correlation of green tea consumption with reduced AD incidents [73]. EGCG exerts neuroprotective effects through multiple mechanisms, including the reduction of APP cleavage and the production of Aβ levels, the inhibition of tau phosphorylation, the regulation of secretase activity, and the acetylcholinesterase inhibition [74,75]. EGCG reduces the aggregation of Aβ peptide and remodels fibrils to form non-toxic forms that lack propagation and prevent cytotoxicity [76,77]. Furthermore, it decreases the oxidative stress by activation of the kelch-like epichlorohydrin-associated proteins (Keap1)/nuclear factor erythroid 2-related factor 2 (Nrf2) signaling pathway [78], inhibits the inflammation by regulation of cytokines (IL-1β, IL-6, and TNF-α), and reduces the NLRP3 inflammasome in microglial cells [79]. In addition, it is highlighted that the activation of EGCG to the gut-brain axis, where EGCG (alone or with genistein) may restore gut microbiota balance, and thus, it suppresses neuroinflammation and resumes autophagy in order to mitigate the AD pathogenesis [80]. A study has shown the EGCG’s ability to restore learning parameters, enhance memory function, increase hippocampal BDNF levels, and minimize hippocampal APP expression in AD models [81]. EGCG improves behavioral and cognitive deficits in late-onset AD, especially when combined with urolithin A, a mitophagy enhancer [82].

### 5.5. Saffron

Saffron (*Crocus sativus* L.) and its bioactive constituents, particularly crocins, trans-crocetin, and safranal, have demonstrated significant potential in the treatment and prevention of AD. Preclinical studies show that saffron reduces Aβ and tau protein aggregation, controls glutamate levels, and minimizes oxidative stress [83], exhibiting multifunctional neuroprotective effects [84]. Saffron’s components, such as trans-crocin-4 (TC4) and trans-crocetin, have demonstrated that they can suppress Aβ aggregation and fibrillogenesis, modulate β- and γ- secretases activity, reduce tau phosphorylation, and suppress kinase activation [85,86]. Added to that, trans-crocetin can also enhance Aβ degradation by increasing the levels of cathepsin B, a lysosomal protease, in AD monocytes [87]. Moreover, in neurodegenerative disease models, TC4 and trans-crocetin exert anti-inflammatory, antioxidant, and anti-apoptotic effects, improving mitochondrial function and controlling metal ion homeostasis [88,89]. In a recent study, isolated components from the stigmas of saffron (*Crocus sativus* L.) induced substantial alteration in the monomer/oligomer distribution of Aβ_1–40_, along with re-direction of fibril formation [84]. This is promising towards the development of novel aggregation inhibitors for the prevention or treatment of AD. In addition, saffron may exert benefits on AD via its anti-diabetic properties, as diabetes is a critical risk factor for AD progression [90]. Furthermore, in vivo studies have demonstrated detection of TC4 in mouse brains for the first time, thus providing preliminary evidence on TC4’s ability to penetrate the BBB, albeit its extremely hydrophilic character [91]. These results are a good indication that TC4 could serve as an active pharmaceutical ingredient and lead compound for developing novel neuroprotective agents. Saffron’s capacity to improve cognition, enhance learning behavior, and prevent hallmark AD pathology supports its application as a neuroprotective agent in clinical practice for the treatment of AD [92]. Clinical trials have indicated saffron’s non-inferiority to standard medications such as donepezil and memantine in improving cognitive function, with fewer side effects [93,94]. Despite the promising findings, further research is needed to elucidate mechanisms, optimize dosing, and develop effective delivery systems [89].

### 5.6. Ashwagandha

Ashwagandha (*Withania somnifera*—WS) has emerged as a promising herbal for the prevention and treatment of AD due to its neuroprotective activity [95]. WS roots are rich in bioactive compounds that exert neuroprotective effects by suppressing Aβ plaque formation and inhibiting tau protein aggregation, reducing oxidative stress, and mitigating inflammation [96,97]. Studies have revealed WS extracts to enhance cognition, memory, and learning while reversing neurological changes such as mitochondrial dysfunction and synaptic loss and boosting neural regeneration [98,99]. Similar results have been concluded in AD mouse models, where administration of WS aqueous extracts has improved spatial memory, decreased depressive and anxiety-like behaviors, reduced Aβ plaque formation, upregulated antioxidant gene expression, and minimized microglia and astrocyte activity [100]. It has been shown that WS and especially its active constituents, Withaferin A, Withanolide-D, Withanolide-E, and Withanolide-G, exert antioxidant and anti-inflammatory responses via regulation of NF-κB and Nrf2 pathways, presenting enzyme inhibition scores higher than the traditional medication drugs [96]. The improved memory and executive function following WS administration have also been confirmed by human studies, indicating its capacity to reveal AD symptoms [101]. Certainly, larger and well-controlled clinical studies are required to validate the effect of this promising drug candidate [102], as most results derived from preclinical studies or trials on healthy individuals [97].

### 5.7. Cannabidiol

Cannabidiol (CBD), a non-psychoactive compound derived from *Cannabis sativa*, has been reported as a promising AD therapeutic with potential through a multifaceted mechanism. Preclinical studies in AD mouse models have shown that CBD and cannabidiol acid suppress pTau and Aβ aggregation, mitigate neuroinflammation and regulate proinflammatory cytokine release, improve synaptic plasticity and reduce ROS formation, while enhancing both short- and long-term spatial memory and cognitive function [103,104,105,106]. Additionally, CBD increases microglia Aβ phagocytosis and mitigates mitochondrial dysfunction via TRPV2 activation, rendering TRPV2 as a potential therapeutic target in AD [107]. CBD has been reported to improve the Aβ cognitive deficit by regulating key proteins such as apolipoprotein E, presenilin-1, and glutamate [108], while it restores the levels of a receptor channel TRPM7 [109] and mitigates the function of inhibitory extra synaptic glycine receptor in hippocampal dentate gyrus [65], alleviating AD’s symptoms. Furthermore, the increased acetylcholine production and changes in ILC2s distribution further underscore the CBD’s therapeutic profile [63]. Combination of CBD with Δ9-tetrahydrocannabinol minimizes cognitive decline in APP/PS1 mice by regulating extracellular glutamate levels and hippocampal hyperexcitability [110]. Despite encouraging in vitro and in vivo findings [111,112,113], clinical data remain sparse, necessitating well-controlled human trials to evaluate safety, pharmacodynamics, and efficacy [114,115]. Overall, CBD represents a compelling avenue for AD therapy, with mounting evidence supporting its role in modulating Aβ and tau pathologies and neurodegenerative mechanisms [116].

## 6. Nanomedicine-Based Delivery Systems for Natural Products

**Figure 2:**
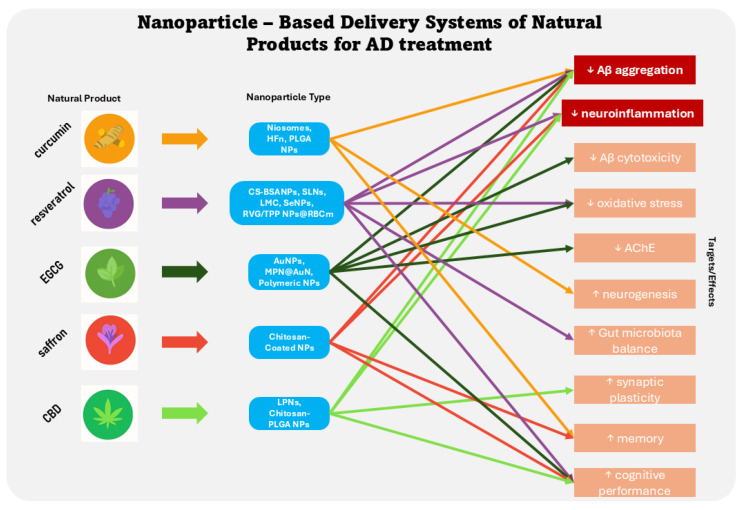
Diagram illustrating the therapeutic targets/effects modulated by selected natural products delivered via nanoparticles for Alzheimer’s disease, highlighting reductions (↓) in pathological features and enhancements (↑) in neuroprotective mechanisms. (EGCG, epigallocatechin gallate; CBD, cannabidiol; HFn, H-Ferritin nanocages; PLGA, poly(lactic-co-glycolic acid); NPs, nanoparticles; CS-BSANPs, chitosan-coated bovine serum albumin NPs, SLNs, solid lipid NPs; LMC, hollow-structured manganese-doped cerium dioxide; SeNPs, selenium NPs; RVG/TPP NPs@RBCm, red blood cell membrane-coated nanostructured lipid carriers bearing rabies virus glycoprotein and triphenylphosphine cation molecules attached to the RBC membrane; AuNPs, gold NPs; MPN@AuN, metal-phenolic network gold nanodelivery system; LPNs, lipid NPs).

Despite the anti-AD properties of numerous natural products, their clinical use has been hampered by pharmacokinetic limitations such as poor bioavailability and limited Blood-Brain Barrier penetration [117]. In recent years, nanoparticle (NP)-based drug delivery systems have emerged as a promising strategy to address these pharmacological challenges of natural products [118]. Various NP types have been investigated for AD, including polymeric NPs, liposomes, dendrimers, solid lipid NPs, as well as magnetic and inorganic NPs [119]. Delivery via polymeric-, lipid-, and protein-based NPs has been reported to overcome key pharmacokinetic limitations of natural compounds such as curcumin and resveratrol, and thus, they effectively inhibit Aβ aggregation, reduce tau phosphorylation, activate neuroprotective signaling pathways, modulate neuroinflammation, and improve cognitive performance [120]. Herein, studies published in 2020–2025 (the research has been performed on the PubMed platform) are presented for curcumin, resveratrol, EGCG, crocin, and CBD (Figure 2).

### 6.1. Curcumin Delivery via Nanoparticles for AD

Numerous NP-based approaches have been explored to enhance curcumin’s therapeutic potential in AD. Studies have shown that curcumin delivered via niosomes modulates NF-κB gene expression present at AD, reduces tissue damage, and improves memory, indicating enhanced efficacy in mitigating AD-related pathology [121]. Niosomes, as efficient nanocarriers, offer advantages in delivering antioxidants more effectively within a shorter therapeutic window. Repeated long-term use of nanomaterials may lead to their accumulation in the body, posing safety concerns, particularly in the brain. Thus, the development of biodegradable, non-toxic NPs capable of crossing the BBB and degrading efficiently is crucial [122]. H-Ferritin (HFn) nanocages as a nanoformulation vehicle for curcumin have shown promising safety profiles and anti-inflammatory effects in both in vitro and in vivo AD models [123]. Besides, polymeric NPs such as poly(lactic-co-glycolic acid) (PLGA)—based curcumin NPs have demonstrated efficacy in various preclinical and clinical settings, yet the potential for toxic effects warrants further investigation [124]. Overall, these findings highlight the promise of nanotechnology in enhancing curcumin delivery for AD, while underscoring the need for careful design to balance efficacy with long-term safety.

### 6.2. Resveratrol Delivery via Nanoparticles for AD

Various NP delivery systems have been developed to overcome the pharmacokinetic challenges of resveratrol in AD treatment. Chitosan-coated bovine serum albumin NPs (CS-BSANPs) have demonstrated safety and efficacy for intranasal brain delivery, improving treatment outcomes in elderly females with AD [125]. In addition, resveratrol-loaded solid lipid NPs (SLNs) enhanced cognitive performance, reduced lipid peroxidation, increased glutathione levels, and preserved hippocampal cell morphology in vivo, outperforming free resveratrol [126]. Other advanced platforms include hollow-structured manganese-doped cerium dioxide NP (LMC), which cross the BBB, reduce oxidative stress, and inhibit Aβ aggregation [127], and B6 peptide-/resveratrol-/oligomeric proanthocyanidin-/hyaluronic acid-grafted (B6-RES-OPC-HA) NPs, which reduce ROS, inflammation, and memory deficits in AD mice [128]. Furthermore, it has been reported that resveratrol-loaded selenium NPs/chitosan NPs (Res@SeNPs@Res-CS-NPs) restore gut microbiota balance, reduce neuroinflammation, and regulate key AD signalling pathways [129]. Similarly, resveratrol-selenium NPs (RSV-SeNPs) and resveratrol-selenium NPs decorated with a Blood-Brain Barrier transport TGN peptide (TGN-Res@SeNPs) exhibit potent antioxidant, anti-inflammatory, and Aβ-inhibitory effects while modulating the gut-brain axis [130,131]. A novel biomimetic delivery system of red blood cell membrane-coated nanostructured lipid carriers bearing rabies virus glycoprotein and triphenylphosphine cation molecules attached to the RBC membrane (RVG/TPP NPs@RBCm) targets neuronal mitochondria, alleviating mitochondrial oxidative stress and improving memory in APP/PS1 mice [132]. Collectively, these nanotechnologies enhance resveratrol delivery, offering multi-targeted approaches to mitigate AD pathology through antioxidant, anti-amyloid, anti-inflammatory, and gut-modulating mechanisms.

### 6.3. EGCG Delivery via Nanoparticles for AD

Various nano delivery systems have also been developed to address the low bioavailability and poor BBB permeability of EGCG. For example, gold NPs (Au-EGCG) effectively inhibited Aβ aggregation and reduced Aβ-induced cytotoxicity in vitro [133]. Similarly, green tea gold NPs (GT-Au NPs) demonstrated inhibition of Aβ aggregation and promotion of fibril disaggregation, highlighting their potential in Aβ modulation [134]. Further advancements include polymeric and glycosylated carbon dot-based EGCG NPs that showed enhanced brain delivery, improved oxidative stress modulation, and reduced acetylcholinesterase activity in the cortex and hippocampus of AD models [135]. A biocompatible metal-phenolic network (MPN) gold nano delivery system (MPN@AuN) composed of EGCG and Zn(II), displayed superior anti-amyloid activity due to its porosity and demonstrated BBB permeability, offering a broad therapeutic scope beyond protein aggregation inhibition [136]. Notably, co-delivery of EGCG with a therapeutic gene (shRNA) in a polymeric NP (REGS-PN) significantly enhanced brain targeting, reduced amyloid burden, and improved cognitive performance in APP/PS1 mice compared to EGCG alone [137]. Together, these studies highlight the therapeutic potential of EGCG nanocarriers in AD by improving delivery efficiency, enhancing bioactivity, and targeting multiple disease pathways. Still, continued research is essential to validate their safety and efficacy in clinical settings.

### 6.4. Crocin Encapsulation for AD Treatment

Low stability and bioavailability of crocin have been attenuated by its encapsulation in chitosan-coated NPs, resulting in significant improvement in memory-related parameters and superior efficacy in mitigating AD-related impairments compared to the same dose of free crocin [138].

### 6.5. CBD Delivery via Nanoparticles for AD

CBD combined with brain-derived neurotrophic factor (BDNF) represents a promising therapeutic strategy for AD, as it mediates both neuroinflammation and neuronal plasticity loss. In another study, has been stated that functionalized lipid NPs (LPNs) co-encapsulating CBD and plasmid BDNF saw high encapsulation efficiency, sustained CBD release, and enhanced cellular uptake across multiple brain cell types, increasing BDNF expression and reducing pro-inflammatory cytokines TNF-α and IL-1β in vitro [139]. Similarly, mannose-conjugated chitosan-coated PLGA NPs (CHTMAN-PLGA) targeting glucose transporter-1 receptors showed a 4-fold increase in BDNF expression compared to naked pBDNF, while exhibiting favorable cytotoxicity and hemocompatibility profiles [140]. Additionally, reduced Aβ plaque burden, upregulated cannabinoid receptors CB1 and CB2, and improved cognitive function have been achieved by chitosan-coated CBD NPs in AD rat models [141]. These findings highlight the potential of brain-targeted nanocarriers co-delivering CBD and BDNF to modulate key AD pathologies and warrant further in vivo validation.

## 7. Clinical Implications and Future Perspectives

The existing treatment options for AD approved by the FDA remain limited. AD is a complex neurodegenerative disorder, and the development of effective therapeutic strategies is crucial for managing symptoms, slowing disease progression, and improving quality of life in individuals with AD. The available pharmacological treatments for AD currently focus on modest symptomatic relief rather than directly targeting the AD underlying neurochemical mechanisms. The most commonly used therapeutic agents are acetylcholinesterase (AChE) inhibitors, whereas there are promising results from ongoing clinical trials employing anti-amyloid monoclonal antibodies (donanemab, gantenerumab, and lecanemab) primarily in early stages of the disease. Nevertheless, AChE inhibitors can alleviate the cognitive symptoms of AD patients, but there is no alteration or delay in the disease progression. On the other hand, second-generation anti-amyloid monoclonal antibodies demonstrate efficacy in early stages of AD and improve cognitive decline, especially in higher doses. Nevertheless, there is no evidence to stop or reverse the progression of AD. Therefore, a wider range of molecular targets that are associated with the AD pathology should be considered to discover potent and safe anti-AD agents. These targets include production and oligomerization of Aβ and Aβ-induced inflammation, as well as activation of microglial cells and their neuroprotective function.

In recent years, medicinal plants have gained attention for evaluating their potential as therapeutic or disease-preventive agents for AD. It should be emphasized that medicinal plants can provide a valuable source of bioactive natural products that can serve as therapeutic or disease-preventive agents for AD. They can also serve as scaffolds for providing synthetic structural analogues of bioactive natural products with improved properties. These natural products show significant clinical potential for AD treatment by targeting multiple pathological pathways of AD, like oxidative stress, Aβ and tau aggregation, as well as other AD-correlated targets such as neuroinflammation. It should be noted that several of these natural products, such as resveratrol, EGCG, *Ginkgo biloba*, saffron, and Traditional Chinese medicine, have demonstrated multi-targeted neuroprotective effects in preclinical studies, targeting important key AD mechanisms like amyloid aggregation and neuroinflammation. In that respect, nutraceutical intervention through specific diets, such as the Mediterranean diet and Asian diet, could be an efficient way to prevent and/or reduce the risk of AD. This has been demonstrated in the HELIAD study, where better cognitive performance and lower dementia rates have been observed for people following the Mediterranean diet [142].

Nevertheless, the clinical application of nutraceuticals has been constrained by poor bioavailability and inadequate BBB penetration. The challenge of drug delivery across the BBB can be efficiently encountered with nanotechnology-based delivery systems. Nanomaterial delivery strategies have achieved improved brain delivery, enhanced therapeutic efficacy, and modulation of key pathological processes inherent to AD, such as Aβ aggregation/inhibition, neuroinflammation, tau hyperphosphorylation, and oxidative stress. In summary, NP-based delivery systems represent a promising tool to unlock the full therapeutic potential of natural products for the treatment of AD. However, there is a need for well-designed medical protocols for the use of nanoparticles for clinical applications in the treatment of AD. In particular, the future use and application of NP-based AD therapies in clinical practice should include the long-term safety, efficacy, biodegradability, and stability of these delivery systems.

Finally, drug repurposing could be a promising avenue for potential AD treatment. This strategy offers a cost-effective and time-efficient approach to identifying potential therapeutic options. A prime example is GLP-1R (Glucagon-Like Peptide-1 Receptor) agonists. Preclinical studies on GLP-1R agonists in AD models have demonstrated promise in reducing Alzheimer’s risk and slowing cognitive decline by reducing inflammation, clearing amyloid and tau, and improving brain insulin sensitivity [143]. Ongoing clinical trials are investigating the potential cognitive benefits of GLP-1R agonists in AD patients, evaluating their role as a therapeutic option for AD.

## Figures and Tables

**Table 1:** Summary of the FDA—Approved Medications for Alzheimer’s Disease ^1^.

Category	Drug (Brand Name)	Mechanism	Approved Use	Comments	Side Effects
Disease-Modifying (Anti-amyloid)	Donanemab (Kisunla™, Eli Lilly, Indianapolis, IN, USA)	Anti-amyloid antibody (IV every 4 weeks)	Early AD (MCI/mild dementia with amyloid confirmed)	Slows cognitive and functional decline	ARIA, infusion reactions, headache, dizziness
Disease-Modifying (Anti-amyloid)	Lecanemab (Leqembi^®^, Eisai Co., Tokyo, Japan)	Anti-amyloid antibody (IV every 2 weeks)	Early AD (MCI/mild dementia with amyloid confirmed)	Reduces cognitive decline, improves function	ARIA, infusion reactions, headache, dizziness
Symptomatic (Cognition)	Donepezil (Aricept^®^, Eisai, Co., Tokyo, Japan)	Cholinesterase inhibitor	Mild to severe dementia (AD)	Stabilizes memory/thinking temporarily	Nausea, diarrhea, insomnia, dizziness
Symptomatic (Cognition)	Galantamine (Razadyne^®^, Janssen Pharmaceuticals, Beerse, Belgium)	Cholinesterase inhibitor	Mild to moderate dementia (AD)	Enhances communication between neurons	GI upset, dizziness
Symptomatic (Cognition)	Rivastigmine (Exelon^®^, Novartis, Basel, Switzerland)	Cholinesterase inhibitor	Mild to moderate dementia (AD & Parkinson’s)	Available in patch form	GI side effects, dizziness
Symptomatic (Cognition)	Memantine (Namenda^®^, Forest Laboratories (Licensed from Merz Pharma), New York, NY, USA)	Glutamate regulator (NMDA receptor antagonist)	Moderate to severe AD	May improve daily functioning	Headache, dizziness, constipation, confusion
Symptomatic (Cognition)	Donepezil + Memantine (Namzaric^®^, Allergan, Dublin, Ireland)	Cholinesterase inhibitor + glutamate regulator	Moderate to severe AD	Combines mechanisms for greater benefit	Mixed side effects from both classes
Symptomatic (Sleep)	Suvorexant (Belsomra^®^, Merck & Co., Rahway, NJ, USA)	Orexin receptor antagonist	Insomnia (mild-moderate AD)	Improves sleep quality	Drowsiness, sleepwalking, impaired alertness
Symptomatic (Agitation)	Brexpiprazole (Rexulti^®^, Otsuka Pharmaceutical, Tokyo, Japan)	Atypical antipsychotic	Agitation in AD dementia	First FDA-approved for agitation in AD	Weight gain, sleepiness, dizziness, black box warning for death risk in dementia psychosis

^1^ According to Alzheimer’s Association (www.alz.org., accessed on 14 July 2025).

**Table 2:** Summary of Natural Products, Mechanisms, and Therapeutic Potential in AD Treatment.

Natural Product	Main Bioactive Compounds	Mechanisms of Action	Reported Effects in AD	Limitations	References
Curcumin	Curcumin (from *Curcuma longa*)	Antioxidant, anti-inflammatory, inhibits Aβ aggregation, reduces tau hyperphosphorylation, enhances neurogenesis via PI3K/Akt-GSK3β/Wnt/CREB pathways	Reduces amyloid plaques, improves cognition and synaptic function in preclinical models	Poor bioavailability; needs novel delivery systems	[11,12,13,14,15,16]
Resveratrol	Polyphenol (from red wine, plants)	Antioxidant, anti-amyloidogenic, anti-inflammatory, activates SIRT1 and PI3K-Akt pathway, crosses BBB	Reduces Aβ and tau pathology, enhances neurogenesis, improves cognition	Low bioavailability, rapid metabolism; delivery optimization needed	[17,18,19,20,21,22,23]
*Ginkgo biloba*	EGb 761 extract	Antioxidant, improves cerebral blood flow, reduces oxidative stress, modulates cytokines, reduces apoptosis	Improves memory, reduces neuroinflammation, enhances mitochondrial function	Variable clinical outcomes: dosing and delivery need clarification	[24,25,26,27,28,29]
EGCG	Epigallocatechin-3- gallate (green tea)	Antioxidant, anti-amyloidogenic, reduces tau phosphorylation, modulates gut-brain axis, activates Nrf2	Decreases Aβ aggregation, improves cognition and behaviour, reduces neuroinflammation	Limited bioavailability, needs advanced formulations	[30,31,32,33,34,35,36,37,38,39]
Saffron	Crocin, trans-crocetin, safranal	Antioxidant, anti-amyloidogenic, anti-inflammatory, enhances Aβ clearance, regulates tau, supports mitochondrial function	Comparable to donepezil/memantine in improving cognition with fewer side effects	Requires optimized dosing, delivery, more clinical trials	[40,41,42,43,44,45,46,47,48,49,50]
Ashwagandha	Withaferin A, withanolides	Reduces Aβ and tau pathology, antioxidant via Nrf2, anti-inflammatory via NF-κB, boosts neurogenesis	Improves learning, memory, reduces plaques, promotes neuroregeneration	Limited human data; larger clinical studies needed	[51,52,53,54,55,56,57,58]
Cannabidiol (CBD)	CBD (from *Cannabis sativa*)	Reduces Aβ, tau, neuroinflammation, enhances synaptic plasticity, modulates TRPV2/TRPM7, improves mitochondria	Improves cognition, reduces neurotoxicity, restores neurotransmitter balance	Sparse clinical data; needs controlled trials	[59,60,61,62,63,64,65,66,67,68,69,70,71,72]

## Data Availability

No new data were created or analyzed in this study.

## Abbreviations

Aβ, beta-amyloid peptide; AD, Alzheimer’s disease; APP, amyloid precursor protein; NFTs, neurofibrillary tangles; EGCG, epi-gallocatechin gallate; CBD, cannabidiol; BBB, Blood–Brain Barrier; CNS, Central Nervous System; NLR, nucleotide-binding domain leucine-rich repeat containing, or NOD like receptors; NLRP3, NLR Family Pyrin Domain Containing 3; TNF-α, tumor necrosis factor-alpha; IL-1β, interleukin-1β; IL-4, interleukin-4; IL-6, interleukin-6; IL-13, interleukin-13; IL-16, interleukin-16; GFAP, glial fibrillary acidic protein; ARIA, Amyloid-related imaging abnormalities; GSK-3β, glycogen synthase kinase-3β; NF-κB, Nuclear Factor kappa-light-chain-enhancer of activated B cells; NPs, nanoparticles; ROS, Reactive Oxygen Species; BDNF, brain-derived neurotrophic factor; AChE, acetylcholinesterase.
